# Relaxation measurements of an MRI system phantom at low magnetic field strengths

**DOI:** 10.1007/s10334-023-01086-y

**Published:** 2023-05-20

**Authors:** Michele N. Martin, Kalina V. Jordanova, Anthony B. Kos, Stephen E. Russek, Kathryn E. Keenan, Karl F. Stupic

**Affiliations:** grid.94225.38 000000012158463X U.S. Department of Commerce, National Institute of Standards and Technology, 325 Broadway, Boulder, CO 80305 USA

**Keywords:** Low field MR, MR standards, Phantom, Relaxation

## Abstract

**Objective:**

Temperature controlled T_1_ and T_2_ relaxation times are measured on NiCl_2_ and MnCl_2_ solutions from the ISMRM/NIST system phantom at low magnetic field strengths of 6.5 mT, 64 mT and 550 mT.

**Materials and methods:**

The T_1_ and T_2_ were measured of five samples with increasing concentrations of NiCl_2_ and five samples with increasing concentrations of MnCl_2_. All samples were scanned at 6.5 mT, 64 mT and 550 mT, at sample temperatures ranging from 10 °C to 37 °C.

**Results:**

The NiCl_2_ solutions showed little change in T_1_ and T_2_ with magnetic field strength, and both relaxation times decreased with increasing temperature. The MnCl_2_ solutions showed an increase in T_1_ and a decrease in T_2_ with increasing magnetic field strength, and both T_1_ and T_2_ increased with increasing temperature.

**Discussion:**

The low field relaxation rates of the NiCl_2_ and MnCl_2_ arrays in the ISMRM/NIST system phantom are investigated and compared to results from clinical field strengths of 1.5 T and 3.0 T. The measurements can be used as a benchmark for MRI system functionality and stability, especially when MRI systems are taken out of the radiology suite or laboratory and into less traditional environments.

**Supplementary Information:**

The online version contains supplementary material available at 10.1007/s10334-023-01086-y.

## Introduction

Increasing efforts are being put towards the development of low field (< 1 T) magnetic resonance (MR) systems. Without the siting limitations of high field MR systems, low field systems allow magnetic resonance to be taken outside of a controlled laboratory or radiology suite and used for a broader range of applications. Low field MR can be used to image the roots of plants in soil [1, 2], detect spoilage of food products [3–5], identify explosives [6] or for emergency room use and bedside patient diagnosis [7–9]. Taking an MR system out of the laboratory or radiology suite removes many environmental controls, such as temperature and noise levels, which can result in measurement variation [1, 4, 5]. To ensure a system is functioning properly and that accurate measurements are being recorded, there is a need for reference materials that are characterized over a wide range of magnetic fields and temperatures.

In the work presented here, precise T_1_ and T_2_ values are measured at 6.5 mT, 64 mT, and 550 mT on NiCl_2_ and MnCl_2_ solutions in the International Society for Magnetic Resonance in Medicine/National Institute of Standards and Technology (ISMRM/NIST) system phantom. Previously, quantitative measurements on the system phantom only included the clinical field strengths of 1.5 T and 3.0 T [10]. Here, we expand this range to include 6.5 mT, 64 mT, and 550 mT, which span the range of current low field research [7, 11–13] and clinical systems such as the Hyperfine Swoop (Guilford, Connecticut, USA), the Promaxo System (Oakland, California, USA), the ViewRay MRIdian (Denver, Colorado, USA), the Synaptive MRI (Toronto, Ontario, Canada) and the Siemens Magnetom Free.Max (Malvern, Pennsylvania, USA). In this study, the T_1_ and T_2_ values of five samples from the NiCl_2_ array and five samples from the MnCl_2_ array of the ISMRM/NIST system phantom are measured at temperatures ranging from 10 °C to 37 °C. These measurements can serve as a benchmark to investigate changes over time and across systems in low field MR experiments, particularly for systems that are operating outside of a controlled laboratory or radiology suite.

## Materials and methods

### Hardware

**Fig. 1:**
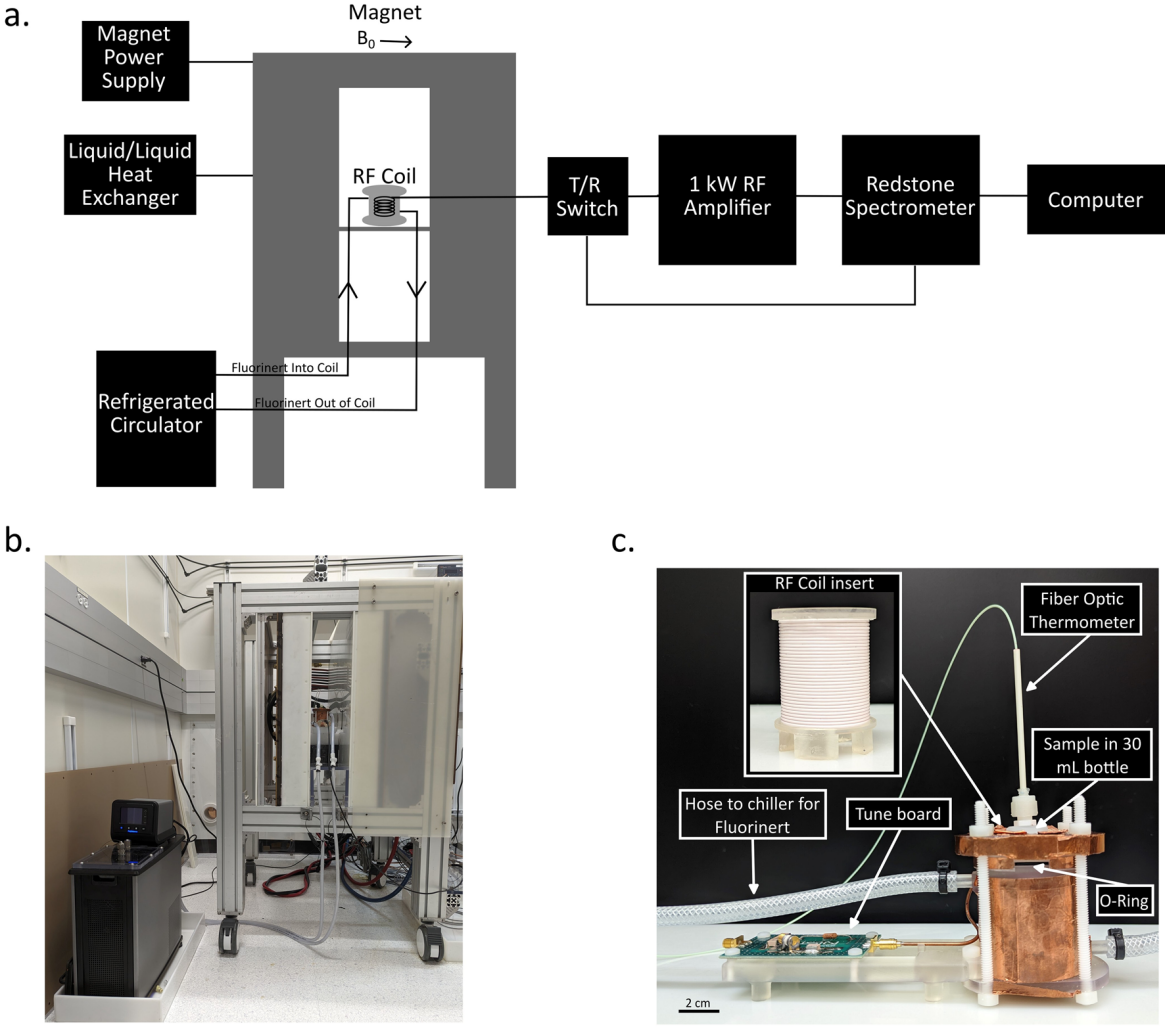
**a** Schematic of the experimental setup. **b** Photograph of the magnet with the RF probe and refrigerated circulator. **c** Photograph of the RF probe

A Redstone spectrometer (Tecmag, Houston, Texas, USA) and Tecmag TNMR software along with a 1 kW RF amplifier (Tomco, Stepney, South Australia, Australia) were used with two variable field electromagnets. The first magnet, an air core magnet (model BFM-0C, Resonance Research Inc., Billerica, Massachusetts, USA), allows for field strengths between 1 and 40 mT. In this study, this magnet, shown in Fig. 1b, was used for relaxation measurements at 6.5 mT (276.5 kHz for ^1^H). The magnet was powered using a magnet power supply (R63C-25265, PowerTen, Kirkland, Washington, USA), cooled by a liquid/liquid heat exchanger (WW2, Haskris, Elmhurst, Illinois, USA) and controlled and monitored using a custom safety system. The second magnet was a 2.9 Ω electromagnet (Bruker, Billerica, Massachusetts, USA) with a pole diameter and spacing of 150 mm and 63 mm, respectively. Operational between 50 mT and 1 T, the electromagnet was used to collect relaxation data at 64 mT and 550 mT (2.73 MHz and 23.4 MHz, respectively for ^1^H). This magnet was powered by a power supply (PSC-4, Walker Scientific Inc., Joondalup, West Australia, Australia) with control voltage inputs with better than 1 ppm stability and was cooled by a Neslab liquid/liquid heat exchanger (System III, Thermo Scientific, Waltham, Massachusetts, USA). A schematic of the experimental set up is shown in Fig. 1a.

### RF Probe

A custom probe containing the RF coil form was designed and 3D printed on a Form 3 printer (Formlabs, Somerville, Massachusetts, USA) using Formlabs clear resin (Fig. 1). The probe was designed to allow Fluorinert (Fluorinert Electronic liquid FC-40, 3M, Saint Paul, Minnesota, USA) [14] to flow around the sample for temperature control. This specific Fluorinert thermal fluid, FC-40, remains a liquid over a wide range of temperatures and is used in many heat transfer applications [15–18]. Importantly, Fluorinert FC-40 does not produce a ^1^H NMR signal as it contains no hydrogen, making it ideal for controlling temperature during MR studies without introducing artifacts. Shown in Fig. 1c, the probe is made up of a 62 mm outer diameter, 54.8 mm inner diameter cup that allows temperature controlled Fluorinert FC-40 to continuously flow around the sample. The outer cup has two ports that connect hoses to the probe. The hoses connect the probe to a refrigerated circulator (PolyScience, Niles, Illinois, USA), used to heat and/or cool the Fluorinert FC-40 and keep the sample at a constant and desired temperature. A lid is clamped down using ¼”− 20 nylon threaded rod, nylon nuts, and O-rings (Oil-resistant Buna-N with 0.18 cm width and 3.15 cm inner diameter and an X-profile oil-resistant Buna-N with 0.18 cm width and 5.69 cm inner diameter) to seal the Fluorinert FC-40 compartment while allowing access for the sample (contained in a 30 mL plastic bottle) to be changed. A hole in the lid allows for an AccuSens fiber optic thermometer (OpSens, Quebec QC, Quebec, Canada) to be inserted into the Fluorinert FC-40, that is flowing in the outer cup, to monitor the temperature throughout experiments. The fiber optic thermometer used in this study has an uncertainty of 0.11 °C, based on the maximum deviation from NIST calibrated Resistance Temperature Detectors (RTDs).

Inside the outer cup is an inner insert that serves as the RF coil former. The removable insert allows for a 36 mm inner diameter, 45 mm long solenoid coil to be wrapped around it. Holes in the outer cup allow for the RF coil to be connected to a tune board via a rigid coaxial cable connected to a SMB connector. To tune the coils to the desired ^1^H Larmor frequencies of 276.5 kHz (6.5 mT), 2.73 MHz (64 mT) and 23.4 MHz (550 mT), two solenoid coils were wound. The coil used for 6.5 mT and 64 mT is a 32-turn solenoid wound using insulated 20 AWG Litz wire (New England Wire Technologies, Lisbon, New Hampshire, USA). An SMB connection allowed for easy exchange of tune boards so that the same RF coil could be tuned to the different resonant frequencies. Due to the low capacitance values required to tune the coil to 550 mT (23.4 MHz for ^1^H), a second coil was made of six turns of 15-gauge solid copper wire separated by 5 mm.

While the temperature of the Fluorinert FC-40 in the outer cup was measured throughout the experiment, the set point of the chiller was determined based on the temperature of the sample. A PT-104 Data Logger thermometer (Pico Technology, St. Neots, Cambridgeshire, UK) was placed in water contained in a 30 mL plastic bottle and the chiller settings needed to achieve sample temperatures of 10 °C, 17 °C, 20 °C, 23 °C, 26 °C, 30 °C, and 37 °C were determined. The maximum difference between the measured temperature of the Fluorinert FC-40 and the water sample was 0.44 °C.

### Samples

The sample set included a subset of the NiCl_2_ and MnCl_2_ arrays from the ISMRM/NIST system phantom [10]. Due to the amount of signal averaging required at low fields and therefore the time required for each experiment, only a subset of the fourteen total components that make up each array in the standard system phantom were analyzed in this work. Samples were chosen to include a broad range of concentrations present in the system phantom. The two extreme concentrations were not included due to difficulty in measuring the short T_1_ of the highest concentration sample and due to the time required to measure the longest T_1_ of the lowest concentration sample. The samples analyzed included five NiCl_2_ solutions from the NIST lending library system phantom (model 130, serial numbers 0133 and 0134, CaliberMRI, Boulder, Colorado, USA) with concentrations of 1.04 mM, 2.52 mM, 5.43 mM, 11.30 mM and 23.30 mM (System phantom spheres NiCl_2_-3, NiCl_2_-5, NiCl_2_-7, NiCl_2_-9, NiCl_2_-11) and five MnCl_2_ solutions with concentrations of 0.03 mM, 0.07 mM, 0.14 mM, 0.28 mM and 0.56 mM (System phantom spheres MnCl_2_-3, MnCl_2_-5, MnCl_2_-7, MnCl_2_-9, MnCl_2_-11).

### NMR experiments—inversion recovery and CPMG

T_1_ was measured using a 20-step inversion recovery pulse sequence with a composite 180-degree pulse [19] and delay times between the composite 180-degree and 90-degree pulse ranging from 6 ms to 6 s. Between each scan, a recovery time greater than 5*T_1_ was required to allow for a return to thermal equilibrium. The composite pulses are used to mitigate the effects of field inhomogeneities and off resonance effects [20]. A custom Python program comprised of Anaconda 3 data science processing packages including scipy, numpy, lmfit, and PyOpenGL was used to fit the data using an unweighted, nonlinear least-squares fitting algorithm. The values of T_1_ were calculated by fitting the phased, integrated real signal (*S*) to

1 $$\mathrm{S}(\uptau ) =\mathrm{ A}(1-\left(1+\updelta \right) {exp}^{-\frac{\tau }{{T}_{1}}})$$

where *τ* is the delay time between the composite 180-degree and 90-degree pulse, *A* is a parameter that refers to the signal intensity at long* τ* times and *δ* is the inversion efficiency. For all experiments, δ was kept over 0.90 by adding resistance to the tuning circuit which reduces the Q factor and ensures properly shaped square pulses. T_2_ values were measured using a 20 step Carr-Purcell-Meiboom-Gill (CPMG) pulse sequence. Fitting the CPMG data to the equation

2 $$\mathrm{S}(\uptau ) = {\mathrm{S}}_{0} {exp}^{-\frac{\tau }{{T}_{2}}}$$

where S_0_ is a fit parameter and *τ* is the delay time between 180-degree pulses, allowed for the calculation of the T_2_ value for each sample.

Forty-eight signal averages were acquired for each scan at 6.5 mT to give a signal to noise ratio (SNR) of 50. Higher SNR at the higher fields allowed for 8 signal averages to be acquired at 64 mT (SNR = 50) and 550 mT (SNR = 250). T_1_ and T_2_ values were measured for all ten samples at field strengths of 6.5 mT, 64 mT, and 550 mT. At each field strength, relaxation measurements were measured at temperatures of 17, 20, 23, and 26 °C. The temperature range was increased for the 2.52 mM and 23.30 mM NiCl_2_ samples as well as the 0.07 mM and 0.56 mM MnCl_2_ samples to include 10, 30 and 37 °C. Due to the time required for each experiment, only two samples from both the NiCl_2_ array and the MnCl_2_ array were chosen for the expanded temperature survey. A high concentration sample and a low concentration sample were chosen from each array to represent the T_1_ and T_2_ behavior at these expanded temperatures. The upper limit of 37 °C was chosen based on the limitations of the 3D printed probe. Each measurement was performed in triplicate, and the mean and standard deviation were calculated. To ensure precise measurements, the number of signal averages was chosen so that the standard deviation was kept under 5%.

## Results

The T_1_ and T_2_ values measured for the five NiCl_2_ solutions, at temperatures ranging from 10 °C to 37 °C, are shown in Table S1 of the Supplemental Information. Similarly, Table S2 in the Supplemental Information shows the measured T_1_ and T_2_ values for the MnCl_2_ array for the same temperature range. In addition to the T_1_ and T_2_ values, standard deviations showing the variation in three trials are given in both Table S1 and Table S2. The relaxation values are dependent on both the magnetic field strength and the sample temperature.

**Fig. 2:**
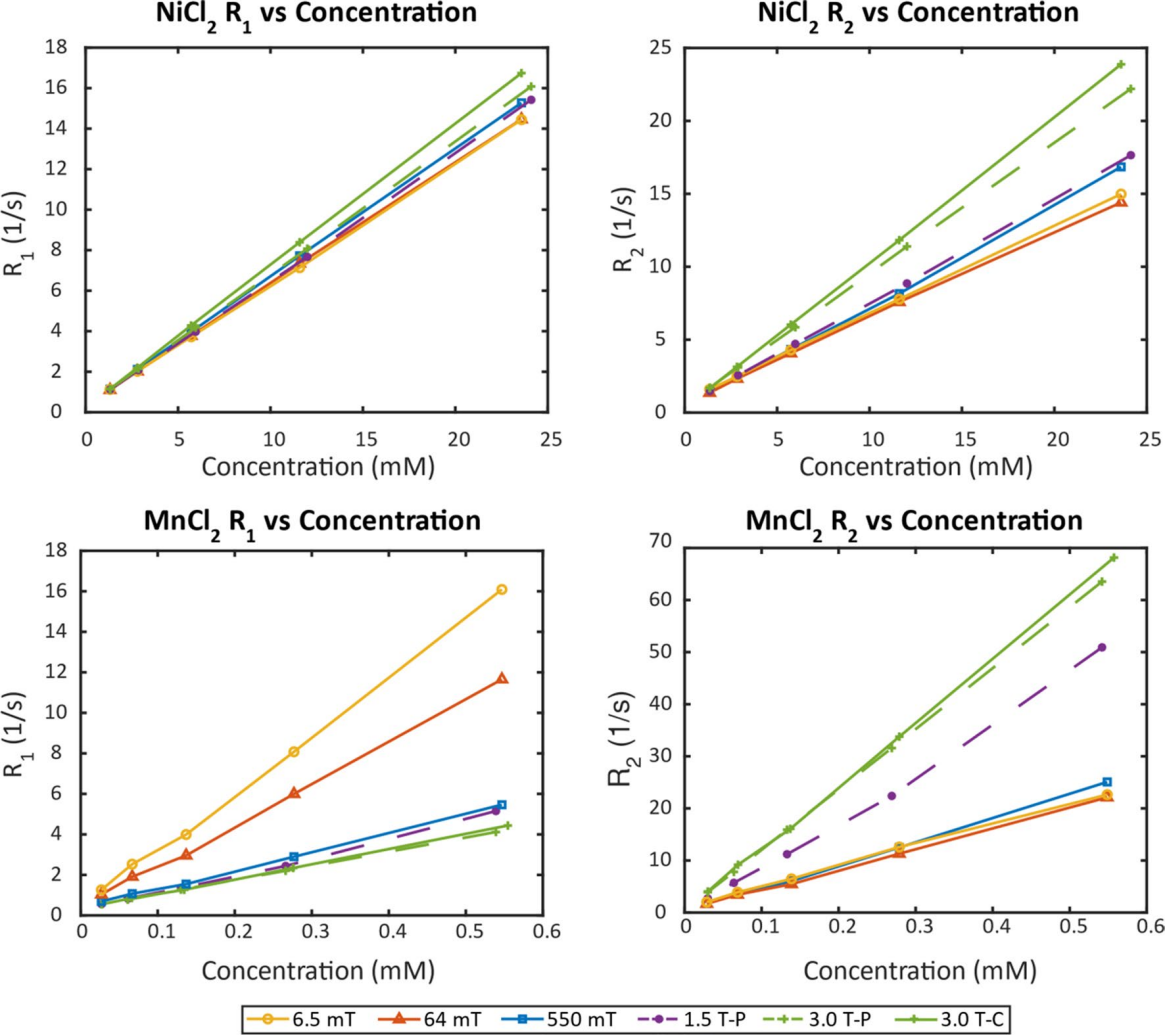
Relaxation rates plotted as a function of paramagnetic ion concentration for NiCl_2_ (top) and MnCl_2_ (bottom) at 6.5 mT (yellow ○), 64 mT (red Δ), 550 mT (blue □), 1.5 T prototype (dashed purple ●) [10], 3.0 T prototype (dashed green +) [10] and 3.0 T commercial (green +). Measurements are taken at 20 °C

In Fig. 2, the relaxation rates (R_1_ and R_2_) are plotted as a function of concentration for both the NiCl_2_ and MnCl_2_ arrays at 20 °C. The temperature of 20 °C was chosen as it is a common room temperature and typical operating bore temperature of many systems. As expected, the relaxation rates increase linearly as the concentration of paramagnetic ions (Ni^2+^and Mn^2+^) increase, resulting in shorter T_1_ and T_2_ values. The 1.5 T-P and 3.0 T-P data is from [10], which are prototype samples that have slightly different concentrations from the ones studied in this work. A second measurement at 3.0 T-C is shown for NIST measurements of the commercial system phantom solutions for the NIST Phantom Lending Library which includes samples with the same concentrations as those used in this low field study. The dispersion of the R_1_ and R_2_ versus concentration lines shows the dependence of the relaxation rates on field for the NiCl_2_ and MnCl_2_ containing solutions.

Based on the data in Fig. 2, the T_1_ relaxivity (r_1_) and the T_2_ relaxivity (r_2_) of the NiCl_2_ and MnCl_2_ arrays were calculated and are shown, along with the ratio of r_1_/r_2_, in Table 1. Relaxivity is a characteristic commonly used in magnetic resonance imaging (MRI) to evaluate contrast agents. The relaxivity indicates the degree to which the relaxation rate of a solution is affected by a paramagnetic species of a given concentration [21]. The r_1_ and r_2_ of the NiCl_2_ solutions shows a small increase of 0.12 and 0.41, respectively, as the field is increased from 6.5 mT to 3.0 T. However, the r_1_ of the MnCl_2_ array decreases by 22.04 as the field is increased, while the r_2_ increases by 80.67 as the field is increased. While both the ratios of r_1_/r_2_ decrease with increasing field, the MnCl_2_ array shows a much more drastic decrease at higher fields. The r_1_/r_2_ of NiCl_2_ decreased ~ 28% from 6.5 mT to 3 T while the r_1_/r_2_ of the MnCl_2_ array decreased by ~ 92%.

**Table 1:** Relaxivity values for each array at different magnetic field strengths at 20 °C

Field (T)	NiCl_2_ r1 (mM^−1^ s^−1^)	NiCl_2_ r2 (mM^−1^ s^−1^)	NiCl_2_ r1/r2	MnCl_2_ r1 (mM^−1^ s^−1^)	MnCl_2_ r2 (mM^−1^ s^−1^)	MnCl_2_ r1/r2
0.0065	0.58	0.59	0.99	28.47	39.62	0.72
0.064	0.59	0.57	1.03	20.46	39.28	0.52
0.55	0.62	0.68	0.92	9.20	44.90	0.20
1.50-P	0.63	0.71	0.88	8.96	93.83	0.10
3.00-P	0.66	0.90	0.73	6.97	116.50	0.06
3.00-C	0.70	1.00	0.70	7.38	121.50	0.06

**Fig. 3:**
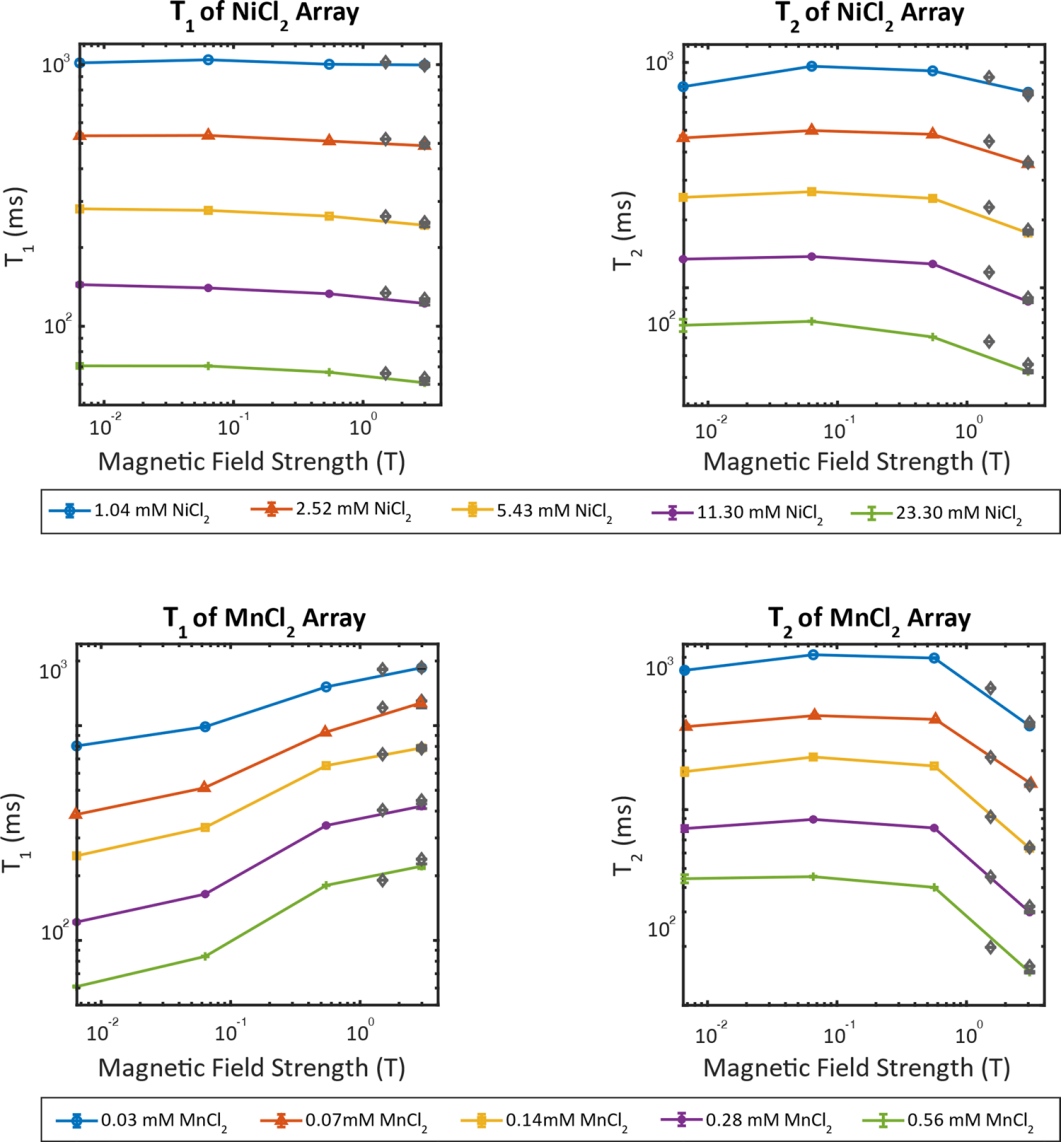
T_1_ (left) and T_2_ (right) as a function of magnetic field strength for the components of the NiCl_2_ (top) and MnCl_2_ (bottom) arrays. Gray ♦ show the measurements of the prototype samples from [10] at 1.5 T and 3 T. All measurements are taken at 20 °C

Figure 3 shows the T_1_ and T_2_ measurements at 20 °C for each sample from both the NiCl_2_ and MnCl_2_ array as a function of magnetic field strength. The plots allow for the visualization of the behavior of T_1_ and T_2_ as the field strength increases from 6.5 mT to 3.0 T. High field data (1.5 and 3.0 T) for the prototype solutions of the system phantom (gray ♦) [10] as well as the values for the commercial system phantom at 3.0 T are added for comparison to the low field data collected in this study. Vertical error bars are included to show the standard deviation of three repeat measurements; for most experiments the error bars are within the width of the plotted data point. While the T_1_ and T_2_ for NiCl_2_ remain relatively constant over the range of field strengths studied here, MnCl_2_ shows a noticeable increase for T_1_ and decrease for T_2_ as the field is increased.

**Fig. 4:**
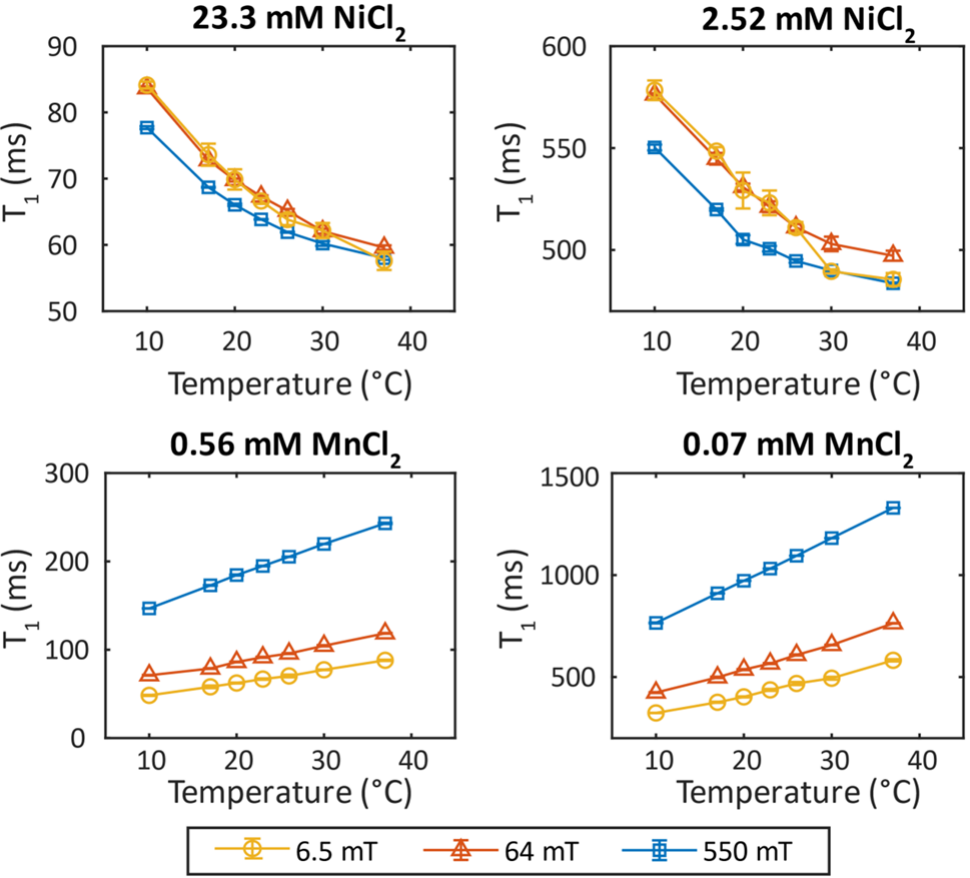
T_1_ as a function of temperature for the 23.3 mM and 2.52 mM NiCl_2_ (top) and 0.56 mM and 0.07 Mm MnCl_2_ (bottom) samples at 6.5 mT (yellow ○), 64 mT (red Δ), and 550 mT (blue □). The error bars show the standard deviation between three trials for each measurement

The T_1_ results for the 23.30 mM and the 2.52 mM NiCl_2_ samples and the 0.56 mM and 0.07 mM MnCl_2_ samples for the expanded temperature range of 10 °C to 37 °C are shown in Fig. 4. The error bars show the standard deviation from three repeat measurements. The NiCl_2_ samples show an exponential decrease in T_1_ as the temperature is increased, which is characteristic of the NiCl_2_ at all concentrations. The MnCl_2_ samples show a linear increase in T_1_ with increasing temperature, again, this behavior is characteristic of all concentrations of MnCl_2_.

Table S3 in the supplemental information shows literature T_1_ and T_2_ values for in vivo human tissue measured at magnetic field strengths at or near 6.5 mT, 64 mT, and 550 mT [22–27].

## Discussion

The enhancement of proton relaxation due to the presence of electron paramagnetic spins has been extensively studied [28]. The increase in the R_1_ and R_2_ (Fig. 2) is linearly proportional to the concentration of paramagnetic compounds in solution and depends on the electron spin-nuclear spin interactions [28]. Ni^2+^ ions cause relaxation in liquid solutions due to small deviations in the octahedral geometry. The small deviations break the symmetry and allow for relaxation rate to be affected [28]. Mn^2+^ ions, on the other hand, have long electronic relaxation times and correlation times which are dependent on molecular tumbling [29].

Comparison of the NMR data presented here to the NMRD relaxometry data in [30] shows a similar trend for both the Ni^2+^ and Mn^2+^ containing solutions. Consistent with the NMRD results for solutions containing Ni^2+^ ions, the small change in the value of r_1_ and r_2_ (Table 1) for NiCl_2_ suggests weak dependence of the relaxation rates on field strength. For solutions containing Mn^2+^, both the NMR data presented here and the NMRD data in [30] show an overall decrease in r_1_ as the field is increased up to 100 MHz [30]. In [30], the r_2_ decreases up to about 10 MHz after which it increases for fields up to 100 MHz. For the field strengths studied here, there is only a slight increase in r_2_ at the lower field strengths of 6.5 mT (276.5 kHz), 64 mT (2.73 MHz), and 550 mT (23.4 MHz) before a steep increase at the higher fields of 1.5 T (64 MHz) and 3.0 T (128 MHz). It should be noted that the pH of the sample can alter the measured T_1_ and T_2_ [31]; therefore, in order to compare to literature values, attention should be given to the pH of the sample. The solutions in this study have a neutral pH of 7 to ensure safety when handling or transporting.

Previous studies have shown no field dependence of the T_1_ of NiCl_2_ solutions at the low fields (< 0.55 T) investigated in this study [29, 31, 32]. The short correlation time, τ_c_, of Ni^2+^ ions that results from fast electron spin relaxation results in no change in the measured relaxation rates as the field is increased up to 2.35 T (100 MHz for ^1^H) [29]. For each of the five components in the NiCl_2_ array, the T_1_ remained constant as the field was increased at low field strengths. As shown in Fig. 3 there is a slight downward trend as the field approaches 3.0 T. The independence of the NiCl_2_ relaxation rates on magnet field strength make the system phantom useful for comparing methods across different MR systems.

On the other hand, the MnCl_2_ shows a marked increase in T_1_ with increasing field (Fig. 3). This change in relaxation rate with magnetic field is explained by the longer electron relaxation times and slower τ_c_. For Mn^2+^ solutions, τ_c_ is determined by the tumbling rate of the ions in solution [29]. Because of the dependence on the ion tumbling rate, the T_1_ of the Mn^2+^ array becomes more dependent on temperature [29]. As shown in Fig. 4, the T_1_ of all components of the MnCl_2_ array increase with increasing temperature. The concentration of Mn^2+^ in the solution affects the degree to which the T_1_ changes because of temperature. At higher concentrations the T_1_ has a more drastic change with temperature. This behavior is seen at all fields. The T_2_ for the MnCl_2_ array shows a smaller change over the temperature range analyzed in this study.

The τ_c_ for Ni^2+^ is more dependent on electron spin relaxation time than ion tumbling causing a decreased dependence of the T_1_ on temperature [29]. As shown in Fig. 4 the NiCl_2_ array shows a decrease in T_1_ values with increasing temperature. The slow decrease as a function of temperature is expected for solutions containing Ni^2+^ ions [32]. In the high field characterization of these phantom components, a benefit of the NiCl_2_ array is that it showed little change in T_1_ and T_2_ at typical bore temperatures (17 °C to 26 °C) at 1.5 T and 3.0 T [10]. However, at the lower fields of 6.5 mT, 64 mT, and 550 mT, the T_1_ and T_2_ values measured between 17 °C and 26 °C showed a constant decrease. This, as well as the MnCl_2_ dependence on temperature, suggests that when the phantom is used on low field systems, accurate sample temperatures should be measured and known.

The ISMRM/NIST system phantom is a stable reference object that can be used to track system stability over time and validate experimental methods. Both the NiCl_2_ and MnCl_2_ arrays span the wide range of in vivo T_1_ and T_2_ values reported in literature for human tissues at low fields (Table S3). While more complex phantoms have been developed that better mimic human tissues [33–38], further study is needed to understand the behavior of those phantoms at low magnetic fields.

The uncertainty in the measurements presented here can be reduced by increasing the homogeneity of both the static and RF fields over the entire RF coil volume. The homogeneity of the static field can be greatly improved by the addition of shims on both low field systems used in this study. Furthermore, the probe, including the RF coil, was designed to be leak-proof so that Fluorinert FC-40 can continuously flow around the sample for temperature control. However, the probe design resulted in the 30 mL bottle samples being large compared to the RF coil volume. Better RF homogeneity can be achieved by redesigning the probe so that the sample is small compared to the RF coil. Finally, the 30 mL bottles containing the samples were not completely full and had empty space at the top. While this portion of the bottle was not in the RF coil volume, it is possible that evaporation during heating may cause the sample in the coil volume to appear more concentrated. In Fig. 4, there is a noticeable drop in the measured T_1_ at higher temperatures at 6.5 mT, this may be explained by a higher than expected sample concentration due to evaporation in the empty space of the bottle. At 6.5 mT, more evaporation may have occurred due to longer experiments required by the necessary, additional signal averaging.

The temperature-controlled, low field relaxation data presented here can serve as a benchmark test to ensure system function and stability. As low field MR systems continue to be developed for industrial and medical systems, which may require use in uncontrolled environments, measurements of standard materials at low magnetic field strengths are important. Further work may be needed to develop a reference object that better mimics the human brain tissue relaxation values at low magnetic field strengths.

## Supplementary Information

Below is the link to the electronic supplementary material.

Supplementary file1 (DOCX 35 KB)

## Data Availability

Data will be made available upon request.
