# Supplementary material for: Relaxation measurements of an MRI system phantom at low magnetic field strengths
Source: MAGMA. 2023 May 20;36(3):477–85. doi: 10.1007/s10334-023-01086-y (PMC10386925; doi:10.1007/s10334-023-01086-y)
Supplement: Supplementary file 1 — Supplementary file1 (DOCX 35 KB) [file 10334_2023_1086_MOESM1_ESM.docx]

Table S1. T_1_ and T_2_ mean and standard deviation measured for the NiCl_2_ array at 6.5, 64 and 550 mT.

| NiCl_2_ | | | | | | | |
| --- | --- | --- | --- | --- | --- | --- | --- |
| Concentration (mM) | **Temperature (°C)** | **6.5 mT** | | **64 mT** | | **550 mT** | |
|  |  | **T_1_ (ms)** | **T_2_ (ms)** | **T_1_ (ms)** | **T_2_ (ms)** | **T_1_ (ms)** | **T_2_ (ms)** |
| 1.04 | 17 | 1039.54 ± 4.89 | 827.3 ± 14.98 | 1024.82 ± 5.64 | 957.08 ± 7.25 | 995.94 ± 0.66 | 915.71 ± 1.50 |
|  | 20 | 1005.43 ± 23.42 | 780.03 ± 4.33 | 1033.18 ± 2.13 | 961.22 ± 6.03 | 993.28 ± 1.22 | 916.67 ± 2.45 |
|  | 23 | 1056.35 ± 12.21 | 807.69 ± 8.39 | 1031.64 ± 2.44 | 962.18 ± 9.20 | 1003.58 ± 3.30 | 924.08 ± 3.59 |
|  | 26 | 1015.44 ± 27.51 | 777.96 ± 8.60 | 1040.24 ± 4.94 | 964.44 ± 5.27 | 1010.15 ± 0.92 | 925.94 ± 1.56 |
| 2.52 | 10 | 578.34 ± 4.82 | 486.19 ± 1.93 | 576.27 ± 0.58 | 539.71 ± 3.27 | 550.30 ± 1.41 | 517.05 ± 3.17 |
|  | 17 | 548.51 ± 1.94 | 466.29 ± 6.55 | 544.74 ± 2.87 | 507.67 ± 0.56 | 519.85 ± 0.59 | 485.82 ± 1.30 |
|  | 20 | 529.18 ± 8.83 | 462.22 ± 10.96 | 530.91 ± 1.65 | 498.49 ± 2.04 | 505.12 ± 2.10 | 479.52 ± 1.24 |
|  | 23 | 523.12 ± 6.05 | 461.24 ± 11.14 | 521.22 ± 2.68 | 487.99 ± 2.28 | 500.62 ± 1.33 | 471.68 ± 4.12 |
|  | 26 | 510.97 ± 2.75 | 430.94 ± 5.73 | 511.15 ± 1.53 | 485.61 ± 1.10 | 494.61 ± 0.87 | 470.05 ± 1.38 |
|  | 30 | 489.52 ± 0.99 | 415.15 ± 18.81 | 502.97 ± 3.57 | 477.78 ± 4.36 | 489.93 ± 0.38 | 469.04 ± 1.70 |
|  | 37 | 485.55 ± 3.12 | 418.83 ± 6.59 | 497.18 ± 2.47 | 466.27 ± 1.69 | 483.67 ± 0.42 | 464.66 ± 3.14 |
| 5.43 | 17 | 283.92 ± 0.69 | 252.17 ± 0.34 | 282.28 ± 0.75 | 274.70 ± 1.20 | 268.16 ± 0.77 | 257.52 ± 0.45 |
|  | 20 | 278.28 ± 7.77 | 251.83 ± 3.07 | 274.54 ± 0.64 | 266.90 ± 0.94 | 260.91 ± 0.11 | 249.21 ± 1.50 |
|  | 23 | 269.19 ± 5.70 | 237.86 ± 1.25 | 262.50 ± 2.43 | 259.89 ± 0.42 | 254.03 ± 0.47 | 243.40 ± 1.50 |
|  | 26 | 259.93 ± 7.89 | 232.6 ± 4.18 | 259.01 ± 1.99 | 252.86 ± 0.38 | 248.93 ± 0.26 | 239.39 ± 1.03 |
| 11.30 | 17 | 148.39 ± 2.57 | 140.74 ± 1.39 | 144.77 ± 0.50 | 143.92 ± 1.25 | 136.67 ± 0.26 | 131.68 ± 0.23 |
|  | 20 | 142.82 ± 1.94 | 134.01 ± 0.54 | 138.70 ± 0.29 | 137.60 ± 0.36 | 131.76 ± 0.14 | 127.54 ± 0.25 |
|  | 23 | 134.59 ± 1.90 | 129.08 ± 1.32 | 134.10 ± 0.35 | 132.54 ± 0.71 | 127.72 ± 0.46 | 123.55 ± 0.62 |
|  | 26 | 130.03 ± 0.89 | 126.6 ± 2.39 | 130.06 ± 0.30 | 128.57 ± 0.88 | 124.74 ± 0.17 | 121.21 ± 0.49 |
| 23.30 | 10 | 84.13 ± 0.52 | 85.98 ± 1.99 | 83.65 ± 0.28 | 84.86 ± 1.05 | 77.70 ± 0.21 | 71.74 ± 0.36 |
|  | 17 | 73.61 ± 1.67 | 73.8 ± 0.37 | 72.79 ± 0.03 | 73.30 ± 0.59 | 68.69 ± 0.05 | 61.96 ± 0.54 |
|  | 20 | 69.88 ± 1.53 | 68.19 ± 4.28 | 69.79 ± 0.22 | 70.98 ± 0.24 | 66.05 ± 0.18 | 60.49 ± 0.08 |
|  | 23 | 66.63 ± 0.44 | 65.62 ± 0.65 | 67.31 ± 0.16 | 68.20 ± 0.86 | 63.85 ± 0.05 | 56.98 ± 0.44 |
|  | 26 | 63.82 ± 0.83 | 63.9 ± 2.92 | 65.15 ± 0.28 | 65.42 ± 0.22 | 61.92 ± 0.07 | 56.43 ± 0.89 |
|  | 30 | 62.16 ± 1.14 | 60.15 ± 2.23 | 62.07 ± 0.36 | 62.36 ± 0.08 | 60.20 ± 0.36 | 55.96 ± 0.16 |
|  | 37 | 57.60 ± 1.39 | 58.93 ± 1.96 | 59.59 ± 0.34 | 59.80 ± 0.40 | 58.00 ± 0.26 | 54.25 ± 0.25 |

Table S2. T_1_ and T_2_ mean and standard deviation measured for the MnCl2 array at 6.5, 64, and 550 mT.

| MnCl_2_ | | | | | | | |
| --- | --- | --- | --- | --- | --- | --- | --- |
| Concentration (mM) | **Temperature (°C)** | **6.5 mT** | | **64 mT** | | **550 mT** | |
|  |  | **T_1_ (ms)** | **T_2_ (ms)** | **T_1_ (ms)** | **T_2_ (ms)** | **T_1_ (ms)** | **T_2_ (ms)** |
| 0.03 | 17 | 761.94 ± 6.38 | 535.59 ± 16.97 | 944.48 ± 4.82 | 609.45 ± 4.86 | 1457.74 ± 5.10 | 582.35 ± 4.81 |
|  | 20 | 822.32 ± 4.32 | 514.14 ± 10.29 | 1010.18 ± 9.68 | 617.54 ± 2.12 | 1548.78 ± 2.30 | 593.85 ± 3.80 |
|  | 23 | 882.74 ± 4.91 | 545.80 ± 9.10 | 1076.87 ± 2.78 | 639.49 ± 12.91 | 1653.48 ± 1.66 | 604.53 ± 3.30 |
|  | 26 | 960.67 ± 10.93 | 528.58 ± 13.32 | 1150.39 ± 9.44 | 620.11 ± 18.80 | 1773.76 ± 6.49 | 610.75 ± 2.93 |
| 0.07 | 10 | 323.77 ± 0.68 | 236.48 ± 9.45 | 423.79 ± 2.14 | 290.02 ± 2.30 | 765.39 ± 2.21 | 263.22 ± 0.57 |
|  | 17 | 376.12 ± 2.54 | 253.91 ± 2.00 | 498.27 ± 3.42 | 298.73 ± 2.24 | 910.71 ± 0.92 | 277.81 ± 0.59 |
|  | 20 | 402.52 ± 2.65 | 260.56 ± 5.60 | 536.33 ± 2.59 | 297.02 ± 3.60 | 972.23 ± 0.44 | 283.87 ± 0.27 |
|  | 23 | 436.87 ± 4.84 | 258.37 ± 6.23 | 566.22 ± 2.47 | 311.84 ± 7.67 | 1031.92 ± 1.11 | 290.62 ± 0.18 |
|  | 26 | 468.28 ± 7.39 | 267.99 ± 11.15 | 608.28 ± 2.59 | 307.45 ± 0.96 | 1094.97 ± 0.43 | 290.10 ± 0.45 |
|  | 30 | 494.03 ± 7.19 | 277.72 ± 8.06 | 657.00 ± 1.38 | 312.50 ± 1.89 | 1181.40 ± 0.68 | 293.21 ± 1.07 |
|  | 37 | 582.00 ± 5.67 | 287.82 ± 8.56 | 763.56 ± 1.20 | 317.42 ± 1.31 | 1329.77 ± 0.62 | 300.53 ± 0.43 |
| 0.14 | 17 | 232.69 ± 10.03 | 151.83 ± 5.66 | 320.10 ± 1.44 | 185.45 ± 1.07 | 624.46 ± 0.19 | 164.53 ± 0.03 |
|  | 20 | 253.31 ± 4.08 | 156.07 ± 7.16 | 343.27 ± 0.53 | 185.37 ± 2.04 | 665.88 ± 0.16 | 166.76 ± 0.17 |
|  | 23 | 264.47 ± 3.98 | 161.15 ± 4.09 | 368.50 ± 1.81 | 186.73 ± 1.11 | 704.59 ± 2.15 | 167.71 ± 0.13 |
|  | 26 | 299.19 ± 4.97 | 162.46 ± 4.59 | 389.42 ± 0.77 | 188.99 ± 1.77 | 743.70 ± 2.02 | 172.29 ± 0.32 |
| 0.28 | 17 | 116.10 ± 1.50 | 77.81 ± 1.38 | 159.31 ± 0.35 | 88.80 ± 0.69 | 329.76 ± 0.83 | 79.40 ± 0.31 |
|  | 20 | 124.50 ± 1.45 | 79.90 ± 2.89 | 168.03 ± 0.65 | 88.98 ± 0.29 | 350.58 ± 2.24 | 80.43 ± 0.30 |
|  | 23 | 130.57 ± 1.07 | 82.62 ± 0.94 | 179.07 ± 1.19 | 88.68 ± 0.17 | 373.64 ± 1.01 | 80.31 ± 0.08 |
|  | 26 | 144.24 ± 1.93 | 84.85 ± 0.57 | 190.57 ± 0.48 | 89.50 ± 2.12 | 395.08 ± 0.96 | 81.83 ± 0.35 |
| 0.56 | 10 | 48.35 ± 0.62 | 41.46 ± 0.57 | 71.09 ± 0.40 | 44.74 ± 0.49 | 146.77 ± 0.14 | 39.01 ± 0.05 |
|  | 17 | 58.01 ± 1.48 | 43.22 ± 0.78 | 78.74 ± 0.40 | 44.18 ± 0.32 | 172.88 ± 0.40 | 40.28 ± 0.07 |
|  | 20 | 62.32 ± 0.50 | 44.30 ± 2.13 | 86.14 ± 0.37 | 45.37 ± 0.26 | 184.57 ± 0.61 | 39.99 ± 0.13 |
|  | 23 | 66.79 ± 0.78 | 44.16 ± 0.46 | 91.46 ± 0.55 | 45.80 ± 0.10 | 194.81 ± 0.29 | 40.56 ± 0.21 |
|  | 26 | 70.32 ± 1.56 | 44.16 ± 1.18 | 95.74 ± 0.50 | 45.34 ± 0.35 | 205.26 ± 0.14 | 40.70 ± 0.20 |
|  | 30 | 77.29 ± 0.37 | 46.50 ± 1.19 | 104.52 ± 0.70 | 45.32 ± 0.30 | 219.76 ± 0.03 | 41.38 ± 0.08 |
|  | 37 | 88.04 ± 0.72 | 45.52 ± 0.34 | 118.58 ± 0.40 | 46.04 ± 0.17 | 243.08 ± 0.76 | 41.85 ± 0.14 |

Table S3. Literature Values with References for human tissues at or near 6.5 mT, 64 mT and 550 mT.

| Field Strength (T) | Tissue Type | T­_1_ (ms) | T_2_ (ms) | Reference |
| --- | --- | --- | --- | --- |
| 0.0065 | Gray matter | 172 | 85 | [21] Sarracanie, Cohen, and Rosen, 2015 |
| 0.0065 | White matter | 127 | 76 | [21] Sarracanie, Cohen, and Rosen, 2015 |
| 0.0065 | Scalp | 91 | 69 | [21] Sarracanie, Cohen, and Rosen, 2015 |
| 0.050 | Gray matter | 327 | 102 | [22] O’Reilly and Webb, 2022 |
| 0.050 | White matter | 275 | 102 | [22] O’Reilly and Webb, 2022 |
| 0.050 | Cerebrospinal fluid | 3695 | 1584 | [22] O’Reilly and Webb, 2022 |
| 0.050 | Calf muscle | 171 | 39 | [22] O’Reilly and Webb, 2022 |
| 0.050 | Subcutaneous and bone marrow lipid | 130 | 90 | [22] O’Reilly and Webb, 2022 |
| 0.064 | White matter (Infant) | 702 | 294 | [24] Artz et al., 2022 |
| 0.064 | Thalamus (Infant) | 364 | 139 | [24] Artz et al., 2022 |
| 0.064 | White matter (cerebellar) |  | 110 | [25] Deoni et al., 2022 |
| 0.064 | Corpus callosum |  | 112 | [25] Deoni et al., 2022 |
| 0.064 | White matter (left frontal) |  | 114 | [25] Deoni et al., 2022 |
| 0.064 | White matter (right frontal) |  | 118 | [25] Deoni et al., 2022 |
| 0.064 | White matter (left posterior­) |  | 98 | [25] Deoni et al., 2022 |
| 0.064 | White matter (right posterior) |  | 102 | [25] Deoni et al., 2022 |
| 0.53 | Colon | 366 | 64 | [26] Koutcher, Goldsmith, and Damadian, 1978 |
| 0.53 | Breast | 554 | 49 | [26] Koutcher, Goldsmith, and Damadian, 1978 |
| 0.53 | Lung | 534 | 92 | [26] Koutcher, Goldsmith, and Damadian, 1978 |
| 0.55 | White matter | 493 | 89 | [23] Campbell-Washburn et al., 2019 |
| 0.55 | Gray matter | 717 | 112 | [23] Campbell-Washburn et al., 2019 |
| 0.55 | Myocardium | 701 | 58 | [23] Campbell-Washburn et al., 2019 |
| 0.55 | Arterial blood | 1122 | 263 | [23] Campbell-Washburn et al., 2019 |
| 0.55 | Liver | 339 | 66 | [23] Campbell-Washburn et al., 2019 |
| 0.55 | Lung | 971 | 61 | [23] Campbell-Washburn et al., 2019 |
| 0.55 | Kidney cortex | 651 | 101 | [23] Campbell-Washburn et al., 2019 |
| 0.55 | Fat | 187 | 93 | [23] Campbell-Washburn et al., 2019 |
